# Machine Learning Classification of Chirality and Optical Rotation Using a Simple One-Hot Encoded Cartesian Coordinate Molecular Representation

**DOI:** 10.1021/acs.jcim.4c02374

**Published:** 2025-05-01

**Authors:** Yilin Zhou, Haoran Zhu, Yijie Yuan, Ziyu Song, Brendan C. Mort

**Affiliations:** Center for Integrated Research Computing, University of Rochester, Rochester, New York 14627, United States

## Abstract

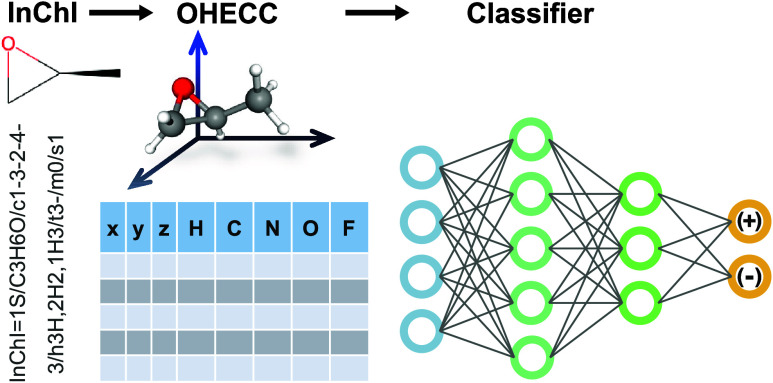

Absolute stereochemical configurations and optical rotations were computed for 121,416 molecular structures from the QM9 quantum chemistry data set using density functional theory. A representation for the molecules was developed using Cartesian coordinate geometries and encoded atom types to serve as input for various machine learning algorithms. Classifiers were developed and trained to predict the chirality and signs of optical rotations using a variety of machine learning methods. These methods are compared, and the results demonstrate that machine learning is a viable tool for making predictions of the stereochemical properties of molecules.

## Introduction

The application of quantum theory to chemistry and the development of quantum chemistry software have demonstrated excellent utility for understanding and predicting experimental observations in chemistry. Historically, attempts to make accurate predictions of molecular structure and properties have been dominated by calculations derived from solutions to various models of the Schrödinger equation for electrons. As models are developed, theoretical predictions of measurable properties have been incorporated into a variety of quantum chemistry software.^1−4^

Chirality of molecules is a fundamental observable in the natural world. While many molecules have the same chemical composition, they can differ in the orientation of bonds around certain atoms. This natural occurrence imparts a “handedness” to the molecular structure for certain classes of molecules that are defined to be chiral. These structural differences have a direct effect on the observable properties of the molecules, namely, their interaction with light, such as the rotation of plane-polarized or circularly polarized light.^5−7^ The direction and magnitude of this phenomenon are known observables for chiral molecules. Theoretical descriptions of this process have been developed that involve differences in electronic transitions induced by left- and right-circularly polarized light.^8^ Much of the theoretical development has led to the implementation of predictions of optical rotation in quantum chemistry software with the goal of making accurate predictions of the interactions between optically active molecules and light. Chiral molecules have an important function in biochemistry and have been shown to be valuable in medicine, including applications as disease biomarkers.^9^

The utility of Hartree–Fock theory in the calculation of the sign and magnitude of the specific rotations of small organic molecules was demonstrated by Polavarapu over two decades ago.^10^ Subsequent developments using time-dependent density functional theory (TDDFT) became (and still remains) a popular method for the practical calculation of optical rotations of small organic molecules and inorganic complexes.^11−13^ It has been shown that absolute configurations can be predicted using quantum mechanical methods.^14^ Additional work has been done to develop more accurate quantum mechanical models using coupled-cluster theory and related approximations.^15−17^ The accurate and reliable prediction of optical rotation still remains an ongoing challenge for chemists despite significant theoretical and methodological advancements.^18^ These calculations are often computationally challenging and require compromises in theoretical models in order to obtain feasible results.

The application of machine learning to a variety of problems in science and engineering has become more common recently due to a number of factors, including the increased availability of data sets, ease of collecting and distributing data, and the advancement of computational hardware and software for performing machine learning tasks. The utility of machine learning in a variety of scientific disciplines has been shown to yield new ways of making accurate predictions. Physics,^19^ Biology,^20^ and other fields have all benefited and continue to advance from applications of machine learning. In 2012, Rupp et al.^21^ made one of the first published attempts to apply a variety of modern machine learning techniques to describe ground-state energies of molecules. Subsequently, machine learning has been applied to predict enthalpy, highest occupied molecular orbital/lowest unoccupied molecular orbital (HOMO/LUMO) gap energy, dipole moment, polarizability, zero point vibrational energy, heat capacity, etc.^22^ Machine learning has been used to predict molecular response properties, such as infrared spectroscopy and polarizabilities.^23^ Improved techniques, including the development of molecular descriptions for common machine learning algorithms, have been developed. Machine learning with experimental data of optical rotations of molecules with a single chiral center from measurements collected in various solvents has been shown to predict the sign of optical rotation with success.^24^ Recently, it has also been shown that machine learning can be used to predict the vibrational circular dichroism spectrum of a molecule from its geometry.^25^ Specialized graph neural networks^26^ have been developed to predict molecular properties and even chirality-aware graph-based neural networks have also been developed to help in the application of QSAR (quantitative structure–activity relationships) in computer-aided drug discovery.^27^

In this work, we demonstrate the application of a variety of machine learning models to the prediction of stereochemical properties of molecules with zero, one, or more chiral centers based on a ground truth developed from density functional theory. We begin with establishing a representation of molecules for machine learning algorithms. While many different representations exist, including those based on force fields, graph theory, and language-based encodings, this work focuses on the use of a representation based on simple Cartesian coordinates, similar to the widely used inputs for quantum chemistry software. These representations, along with their classification labels, are used to train various machine learning models. We report on the validation of these models and give an outlook for future work and the utility of using machine learning to make stereochemical property predictions.

## Theory

### Machine Learning

Machine learning is a subset of artificial intelligence and emphasizes the process of training a mathematical model with data and its appropriate classifications or outputs. Typically, a model is presented with many examples and finds statistical structure in the data and corresponding labels or outputs that eventually allows the system to develop rules for accurately labeling or predicting the output of data that have not been encountered.

In this work, a few common, relatively simple machine learning algorithms are used to analyze a set of data based on information contained in the geometric coordinates of atoms and their types in molecules. Machine learning algorithms commonly used in applications to data problems in science and engineering include random forest (RF), gradient boosted decision trees (GBDT), and artificial neural networks (ANN). These methods can all be used to solve the supervised learning problem, where the model parameters are minimized from some loss function across a set of training data.^28^ Given known features ***x***^(*i*)^, labels ***y***^(*i*)^, and model parameters **θ**, we can optimize for these parameters with
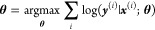
1

This equation is derived from the maximum likelihood estimator for a function and formalizes the supervised learning process.^28,29^ The output of this equation is a set of optimized model parameters based on the features and labels provided from the data set used to train the machine learning model.

#### Random Forest (RF)

Random forest (RF) is a very powerful machine learning algorithm and consists of multiple random decision trees. It is a well-known and useful ensemble method for a variety of classification and regression problems and can provide high accuracy through cross-validation.^30^

**Figure 1:**
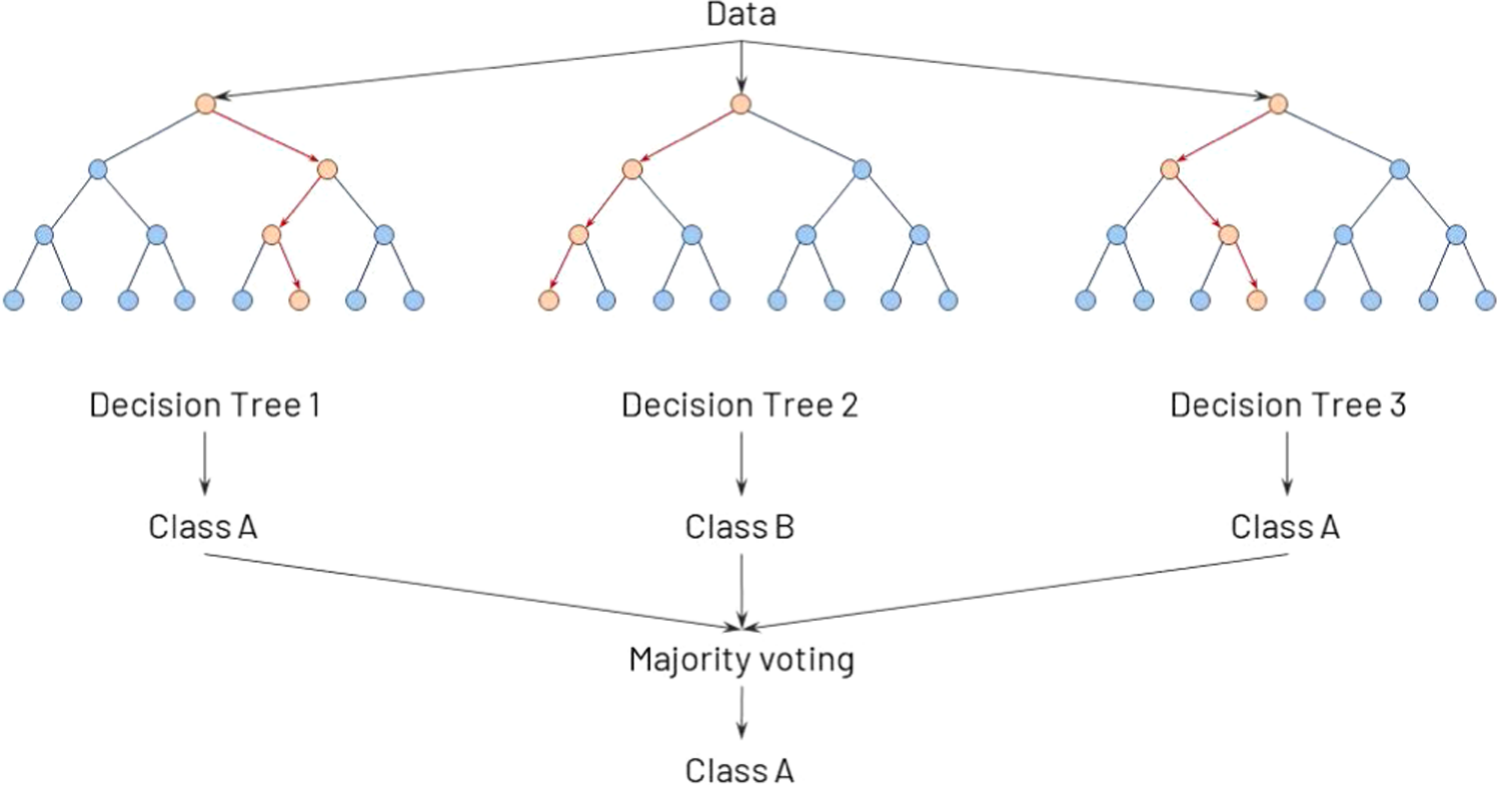
Random forest demonstration

Random forest is an ensemble of *k* trees {*T*_1_(*X*), ···, and *T*_*k*_(*X*)}, where *X* is a set of features used for the machine learning model. The ensemble produces *k* outputs {*ŷ*_1_ = *T*_1_(*X*), ···, *ŷ*_*k*_ = *T*_*k*_(*X*)}. A final prediction *ŷ* is produced from the aggregation of the trees. For classification problems like the ones that are considered in this work, *ŷ* is the class predicted by the majority of trees.^31^Figure 1 demonstrates the algorithm. Each decision tree generates a classification of the input, these results are cast into a vote, and the majority vote is the final classification. In RF, each decision tree is constructed from a random sample of the original data, and at each tree node, a subset of features is randomly selected to generate the best split with the given parameters. The final prediction of any label can be expressed by the following equation:
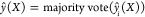
2where *ŷ*_*i*_(*X*) is the class prediction of the *i*th random forest tree.

#### Gradient Boosted Decision Trees (GBDT) and XGBoost

Boosting is where multiple simple weak models (decision trees in this case) combine to generate a stronger model collectively. With gradient boosting, these new models are constructed from gradient descent. Gradient boosting sets targeted outcomes for the next iterative model to minimize errors. Targeted outcomes for each step are based on the gradient of error with respect to the prediction.

XGBoost (extreme gradient boosting)^32,33^ is a high-performance gradient boosted decision tree machine learning algorithm that works well with structured data. Compared with gradient boosting, it adds regularization to the loss function. It is also faster by integrating parallel processing and tree pruning. At the *t*th iteration, the loss function *L* is minimized
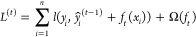
3

where *l* is a differentiable convex loss function that measures the difference between the target *y*_*i*_ and the sum of *f*_*t*_ and the prediction of previous iteration, and Ω is a penalizing term for the complexity of the model. Once the model is optimized, it can be used for inferences. The GBDT machine learning method is implemented in parallel using the XGBoost library.

#### Artificial Neural Networks

An artificial neural network (ANN) is an interconnected group of nodes designed to simulate the way the human brain analyzes and processes information.^34^ The network consists of connections (like the synapses in a biological brain), with connections providing the output of one neuron as an input to another neuron. Each connection is assigned a weight that represents its relative importance. Deep feed-forward neural networks, which consist of two or more hidden layers, are the quintessential deep learning models.^28^ Mathematically, an ANN can be described as a nested function corresponding to various layers in the network:
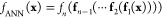
4

Each of the values of the subsequent layers is computed as a function of the previous layer.
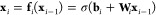
5where σ represents the activation function, which is usually a nonlinear function (e.g., sigmoid, hyperbolic tangent, etc.). The components of these layer functions include weights (**W**_*i*_) and biases (**b**_*i*_).

Typically, the features for each observation are expressed as the initial input vector **x**_0_. The goal of training a neural network is to find the best weights and biases that minimize the difference between the known features and the predicted features. This process requires the repeated calculation of forward propagation and back-propagation through the neural network to find the terms that provide the lowest error.

The loss function is often represented by cross-entropy loss or mean-squared loss. For cross-entropy loss
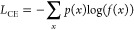
6where *p* is the true probability of *x* and *f* is the network output. For mean-squared error loss, we have
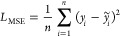
7where *y*_*i*_ is the true class of the sample and *ỹ*_*i*_ is the network output class.

### Time-Dependent Density Functional Theory

In order to build a substantial data set that contains values for optical rotation, including both signs and magnitudes, traditional quantum chemistry software must be used. The most common method for implementing the calculation of optical rotation is time-dependent density functional theory (TDDFT). Beginning with the time-dependent Schrödinger equation
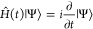
8a Hamiltonian can be constructed to represent a perturbing parameter or multiple parameters. In the calculation of optical rotation, the perturbing field with a frequency ω is provided by electric field  and magnetic field  components.

TDDFT can be useful for providing a theoretical framework for calculating a variety of properties for the corresponding perturbing fields. In the case of optical rotation, the equations can be solved to produce a tensor representing the response of the molecule to the perturbing electromagnetic field
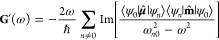
9where **μ** is the electric dipole moment operator and **m** is the magnetic dipole moment operator. The **G**′ tensor can be used to calculate the more familiar specific rotation [α]_λ_ for a molecule at a given wavelength of light. The optical rotation parameter β is readily calculated from the diagonal elements of the **G**′ tensor.
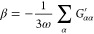
10

The specific rotation [α]_λ_ is directly related to β
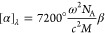
11where *N*_A_ is the Avogadro constant, *c* is the speed of light, and *M* is the molecular weight of the molecule. These equations can be implemented in traditional quantum chemistry software using density functional theory.^11,35,36^

## Computational Details

### Selection of Molecules

In order to examine the chiral properties and optical rotations of molecules, a data set must be chosen that provides a good representation of many different types of molecular structures. The QM9 data set provides a source of organic molecules containing up to 9 heavy atoms (defined as C, O, N, or F). Since chiral information is not directly encoded in the QM9 data set, all 133,885 molecules from the InChI representations in the reference data set were processed with RDKit’s MolFromInchi and FindMolChiralCenters functions to label the number and type of chiral centers (https://www.rdkit.org). From the outputs of the program, 12,230 molecules were not successfully identified for at least one of the potential chiral centers. The Cartesian coordinates of the remaining 121,655 molecules were further processed with Gaussian 16. Out of this set, successfully converged geometry optimizations were obtained for a total of 121,416 molecules using the B3LYP hybrid density functional^37−40^ and the 6–31G** basis set.^41−43^ The final set of molecules can be divided into subsets that represent the number of chiral centers found in each molecule. In a small number of cases, where the chirality is attributed to the intrinsic structure of the molecule, the molecule is classified as having a single chiral center to represent that there are two possible configurations (i.e., clockwise and counterclockwise). Table 1 shows the total quantities for each subset. Each classification is represented notationally as a set of molecules, where, for example, {0} represents the set of all molecules with no chiral centers and {1} represents the set of all molecules with one chiral center. A representation of {0 and 1} would indicate the union of the set of molecules with zero and one chiral centers.

**Table 1:** Cardinality of the Classes of Molecules Corresponding to the Number of Chiral Centers in the Subset of QM9 Molecules Analyzed for This Study

class	number of molecules
{0}	36,175
{1}	22,973
{2}	25,702
{3}	19,880
{4}	11,533
{5}	3,883
{6}	1,110
{7}	134
{8}	26

### Molecular Representation

A matrix with 27 rows and 8 columns was constructed for each of the 121,416 molecules. Each row of the matrix stores the Cartesian coordinates of the atom and the one-hot encoded atom type. The maximum number of rows was set to 27 to correspond to the molecule that has the largest number of atoms in the subset of the QM9 data set that was used in this study. Row entries for molecules with less than 27 atoms were padded with zeros. Three columns were used for the Cartesian coordinates of the molecules, and the other 5 columns were used for one-hot encoding of the atom types (H, C, N, O, and F) for the molecules used in this work. For simplicity, we refer to this representation of a molecule as the one-hot encoded Cartesian coordinate (OHECC) representation.

### Optical Rotation Calculations

The optical rotations of the 121,416 molecules were calculated using Gaussian 16.^1^ The CAM-B3LYP range-separated hybrid density functional^44^ along with the 6–31G** basis set was used to calculate the optical rotations of each molecule at 3 separate wavelengths (355, 589.3, and 633 nm). Optical rotation values from 589.3 nm were used for all machine learning algorithms, and the values obtained from all three wavelengths were used for analysis of calculated optical rotation distributions. The values obtained have been included in the data set and is the first known published data set to our knowledge that includes optical rotation calculations for over a hundred thousand different molecules with optimized geometries provided by DFT. Data for this work is provided by the newly constructed OR-QM9 data set.^45^ It contains the following structure: molecule index number; InChI representation; OHECC representation; number, location, and type of chiral center; and calculated optical rotation at three wavelengths.

### Machine Learning

In this work, we examine six classifications of molecules (five binary and one multilevel) based on three machine learning models. Specifically, we are interested in determining how well machine learning models can predict the existence and number of chiral centers, the appropriate (R) and (S) labels as defined by the well-known Cahn-Ingold-Prelog (CIP) system,^46^ and the sign of the optical rotation of a molecule at the standard 589.3 nm sodium-D line transition frequency. Specifically, we define the following six classifications:

- {0} (zero chiral centers) vs {1, 2, 3, 4, 5, 6, 7, 8} (at least one chiral center) binary classification: The purpose of this classification is to determine whether a chiral center exists in a given molecule. Since the data set has 23,185 molecules with at least one chiral center and 36,247 molecules with no chiral centers, we selected 23,000 molecules from each class to form a new set of 46,000 molecules in order to keep the number of labels balanced in accordance with good sampling methods.
- {0} vs {1} binary classification of a molecule with 0 or 1 chiral centers (0 vs 1 chiral center): This classification compares the effectiveness of being able to label molecules with a single chiral center from other molecules that do not have a chiral center. To avoid a data imbalance problem, 22,500 molecules are randomly sampled from each class.
- Binary classification of the molecules in set {1} partitioned into nonoverlapping sets {R} and {S}, where {R} represents all molecules with one chiral center and an (R) configuration, and {S} represents all molecules with one chiral center and an (S) configuration ((R) vs (S)): This classification problem determines the effectiveness of machine learning for labeling chiral centers with the Cahn-Ingold-Prelog (CIP) rules.
- Binary classification of the molecules in set {1} partitioned into nonoverlapping sets {+} and {−}, where {+} represents all molecules calculated to have a positive optical rotation at 583.3 nm, and {−} represents all molecules calculated to have a negative optical rotation at 589.3 nm. (+ vs −): With this data set, we can determine the accuracy of classifying molecules with a single chiral center as dextrorotatory or levorotatory based on optical rotations at a wavelength of 589.3 nm.
- Binary classification of the molecules in the set {0, 1, 2, 3, 4, 5, 6, 7,8} partitioned into nonoverlapping sets {+} and {−}, where {+} represents all molecules calculated to have a positive optical rotation at 583.3 nm, and {−} represents all molecules calculated to have a negative optical rotation at 589.3 nm. (+ vs – for all): This case is an extension of the previous binary classification, and includes all molecules used in the machine learning study derived from the QM9 data set.
- {0} vs {1} vs {2} vs {3} vs {4} Multilevel classification of a molecule with 0, 1, 2, ···, 4 chiral centers: This multilevel classification allows the machine learning algorithm to determine the number of chiral centers in a molecule. Note that 10,000 samples are randomly chosen from each class, and that the limited data set only allows up to 4 chiral centers due to the number of molecules available for training.

A standard 80–20% training-validation split was employed for each of the data sets used for machine learning. The built-in RandomForest library implemented in Scikit-learn version 0.23.2^47^ and XGBoost as implemented in the xgb Python package^33^ were used to train the respective models. Deep learning algorithms for the ANN models were implemented with PyTorch version 1.8.1+cu102 (Python version 3.9.5)^48^ with GPU acceleration as provided by Nvidia GPUs. All other data preprocessing and postprocessing tasks were completed with the libraries available in version 0.23.2 of Scikit-learn. Statistical analysis of data was performed with the 1.5.4 version of scipy. The average training time for the random forest classification took less than 1 min, while the XGBoost algorithm took several minutes to complete. Each neural network training required approximately 3 or 4 min of computing time on a single GPU. Using grid searches for each model, parameters for random forest models are set as {min_samples_leaf = 2, max_features = “sqrt”}, and other case-specific parameters are listed in Table 2. For XGBoost, the parameters are set as {max_depth = 20}, with other parameters listed in Table 3. For reference, Table 4 shows the grid search results for + vs – classification for {1} with different tree depths for random forest and XGBoost. Different random states result in slightly different accuracies and *F*_1_ scores, but in general, the models provide similar results when the depth is larger than a certain threshold. The values for the number of estimators and the maximum depths of the trees were selected based on the output of the optimal results automatically provided by the grid search.

**Table 2:** Training Parameters for Random Forest Models

chiral property	molecular classes	parameters
chiral center existence	{0, 1, ···, 8}	n_estimators = 1800
		max_depth = 100
0 vs 1 chiral center	{0, 1}	n_estimators = 1200
		max_depth = 420
number of chiral centers	{0, 1, ···, 4}	n_estimators = 1800
		max_depth = 100
(R) vs (S)	{1}	n_estimators = 1800
		max_depth = 100
+ vs –	{1}	n_estimators = 1200
		max_depth = 100
+ vs – for all	{0, 1, ···, 8}	n_estimators = 1200
		max_depth = 420

**Table 3:** XGBoost Parameters

chiral property	molecular classes	parameters
chiral center existence	{0, 1, ···, 8}	default
0 vs 1 chiral center	{0, 1}	default
number of chiral centers	{0, 1, ···, 4}	default
(R) vs (S)	{1}	default
+ vs –	{1}	eta = 0.13
+ vs – for all	{0, 1, ···, 8}	eta = 0.1
		eval_metric = “error”

**Table 4:** Random Forest and XGBoost with Different Depths for + vs – for {1}

random forest			XGBoost		
depth	test accuracy	test *F*_1_ score	depth	test accuracy	test *F*_1_ Score
5	0.5869	0.6718	5	0.6373	0.6619
50	0.7140	0.7338	10	0.6988	0.7157
100	0.7150	0.7345	20	0.7158	0.7298
280	0.7150	0.7345	30	0.7128	0.7257
420	0.7150	0.7345			

**Figure 2:**
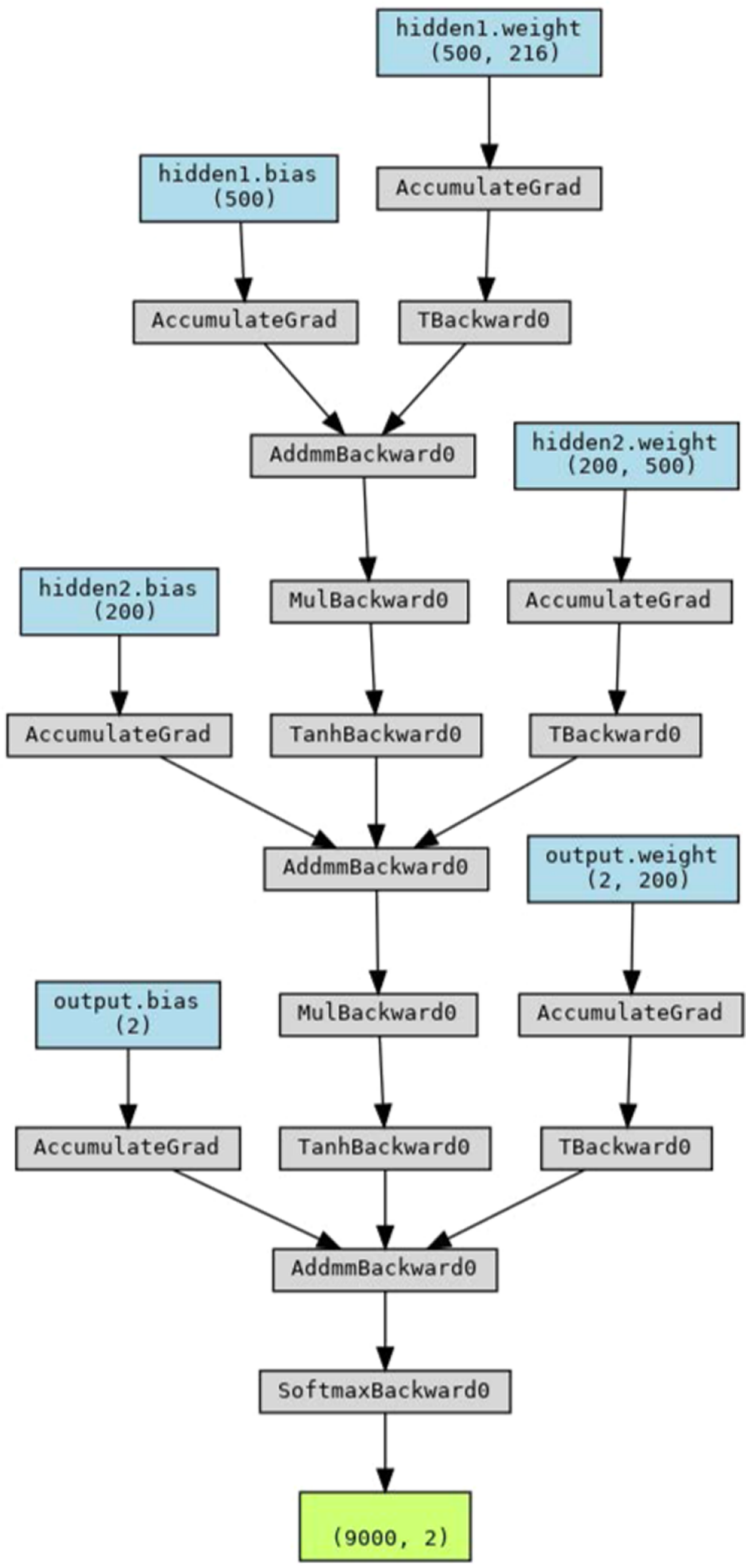
Neural network structure used for training models.

For studies using neural networks, each OHECC matrix (27 × 8) was flattened to an array with length 216. A feed-forward artificial neural network (ANN) was built based on PyTorch with 2 hidden layers of size 500 and 200, respectively, between the input and output layers. The ANN used AdamGrad() as the gradient descent algorithm and CrossEntropyLoss() as the loss function. A dropout rate of 0.05 was employed at each hidden layer. At the output layer, a softmax function was used to classify the output, based on the number of classes in each task. For each training, the network was trained for at least 10,000 epochs until convergence. Figure 2 shows the structure of the neural networks as produced by PyTorchViz visualization package.^49^

For each one of the classification models, a confusion matrix was generated to calculate the accuracy, recall, precision, and *F*_1_ score to analyze the relative performance of the machine learning models. For all confusion matrices, predicted values are the column labels, and the row labels are the actual values. Recall is the fraction of true positives with respect to the total number of true positives and false negatives and attempts to provide insight into the proportion of actual positives that are identified correctly. Precision is the fraction of true positives with respect to the total number of true positives and false positives and attempts to give insight into the proportion of positive identifications that are actually correct. The *F*_1_ score is the harmonic mean of the precision and recall and can be used to compare the relative accuracy of different machine learning models. The range is from 0 to 1, with 0 indicating no precision or no recall and 1 indicating both perfect precision and recall.

## Results and Discussion

### Data Representation

Some of the first machine learning work for predicting properties of molecules made use of Coulomb matrices (CM) for inputs to the algorithms.^21^ These molecular representations essentially provide a table of force fields between each pair of atoms within the molecule based on atomic charge and distance. For any atom pair *i, j*, the element *M*_*i, j*_ of the Coulomb matrix is calculated as
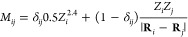
12

The size of the Coulomb matrix matches the number of atoms in the molecule. Rupp et al. have shown the good utility of CM representations for the purpose of predicting molecular properties. Studies have been performed to show that accurate energy predictions and more sophisticated predictions such as excitation spectra are all possible through various machine learning algorithms and CM representation of molecules.^50^

In this work, a simple one-hot encoded Cartesian coordinate representation (OHECC) is introduced. It contains three columns representing Cartesian coordinates of every atom in the molecule and additional columns representing each of the one-hot encoded element symbols. Since the elements of the molecules in this work are only one of 5 different types (H, C, N, O, and F), five columns representing the one-hot encoded element symbol were used consistently for the representations for each of the molecules in the QM9 data set. In this way, each row consists of exactly 8 features. This representation is easy to create and works well with existing representations of molecules commonly used in computational chemistry software. Although the representation is not necessarily unique (similar to a generic Cartesian coordinate representation), a simple coordinate transformation can translate one representation of a molecule into another. It is possible to extend the representation to additional atom types, but it has been limited here based on the molecules used to complete this study.

**Figure 3:**
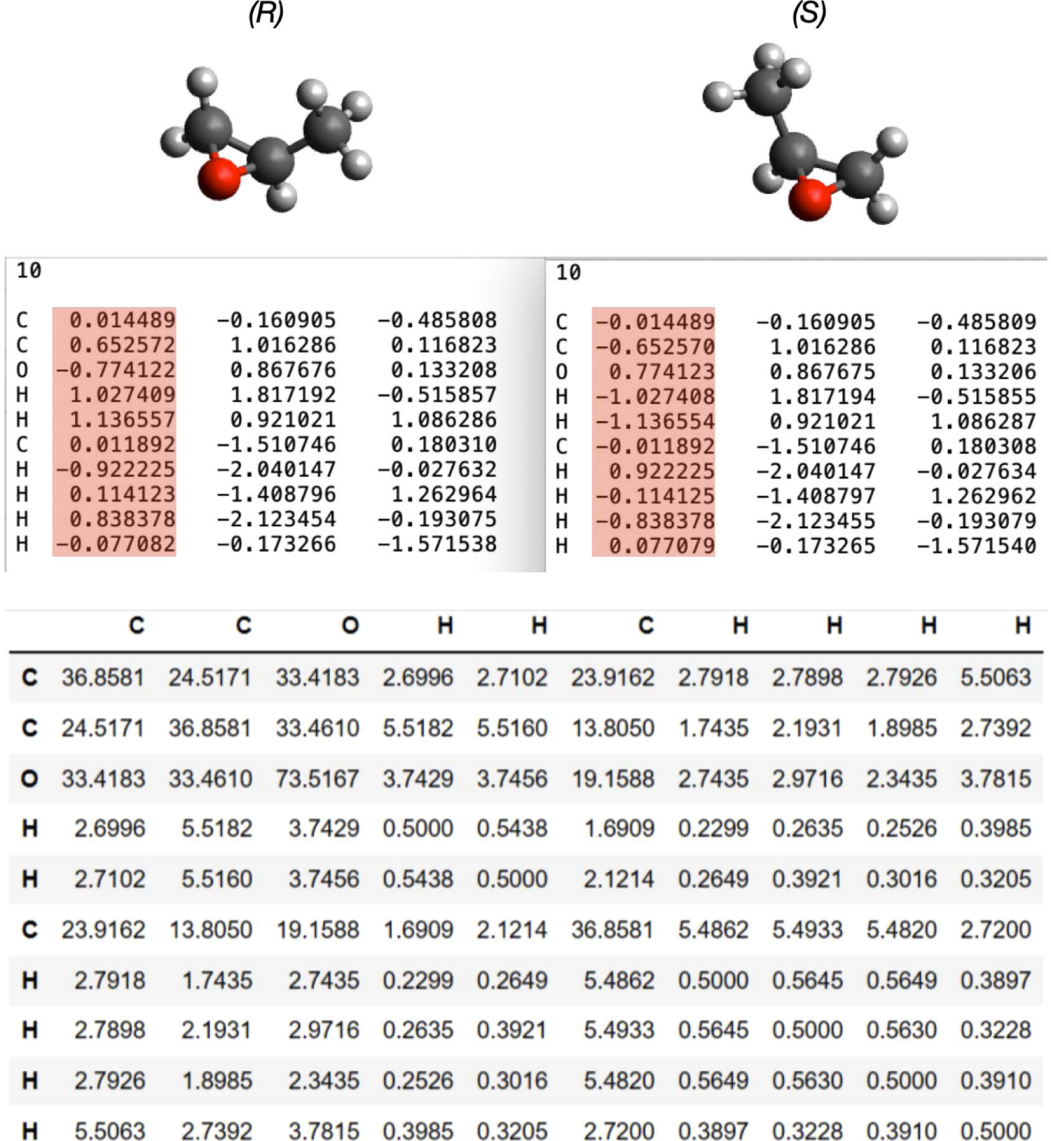
Comparison of the xyz files and Coulomb matrix for (*R*)-methyloxirane and (*S*)-methyloxirane.

The OHECC representation is desirable for distinguishing molecules with similar numbers and types of atoms and connectivity. Unlike the Coulomb matrix and other graph-based representations, zero changes in atom connectivity coupled with spatial reconfiguration of a relatively small number of atoms in a molecule can provide a sufficient resolution of information to aid in machine learning training. Changes in the Coulomb matrix representations between two enantiomers are undetectable due to the isometric transformation that can be used to convert a molecule to its mirror image. However, changes in the OHECC representation are evident because of the absolute arrangement of the atoms in space. Cartesian coordinates can elucidate the isometric transformation between the two isomers. As a result, the features of the OHECC representation provide the appropriate detail for these algorithms to label the configuration accurately. As an example, Figure 3 illustrates the similarity between the Coulomb matrices of the (R) and (S) enantiomers of methyloxirane. Since the two enantiomers are represented by the same matrices, a different approach for representing the molecular structure is required.

The simplified molecular-input line-entry system (SMILES) representation^51^ is a specification in the form of a line notation for describing the structure of chemical species using short ASCII strings. SMILES has been used to predict molecular properties, either through natural language modeling, or transformation into feature matrices followed by machine learning.^52,53^ The (spatial orientation) chirality information in SMILES is represented by an “at” symbol, but this can be removed from the representation if there is no need to distinguish between different arrangements around a chiral center.

The International Chemical Identifier^54^ (InChI) is a textual identifier for chemical substances. Stereochemical information is represented through the t/m/s layer of the string. In general, the InChI expresses the configuration of a stereogenic center or bond as either +/- or m0/m1. These marks have no relation to (R)/(S) tetrahedral or (E)/(Z) alkene configurations. For example, (R)-methyloxirane and (S)-methyloxirane are represented as InChI = 1S/C3H6O/c1–3–2–4–3/h3H,2H2,1H3/t3-/m1/s1 and InChI = 1S/C3H6O/c1–3–2–4–3/h3H,2H2,1H3/t3-/m0/s1, respectively. The InChI strings are different, but there is no association between these strings and the CIP identifiers (R) and (S). In the QM9 data set, although there is no correlation between (R)/(S) identifiers, the InChI entries contain stereochemical information, and it is processed by RDKit^55^ to derive the absolute configurations based on the CIP rules.

Pinheiro et al. identify the importance in data-driven approaches for machine learning and differentiate coordinate distance-based methods from text strings/grammar-based methods for describing molecules used as inputs to machine learning.^52^ One of the benefits of coordinate distance-based approaches is that the local (and often chemically important) structure of a molecule is emphasized. Since enantiomers have different labels (e.g., (R)/(S) or +/-), the differentiation between these molecules is often localized by the configuration of a few atoms or moieties around a chiral center. Furthermore, it may be conceptually desirable to maintain the simplicity of the models (e.g., avoiding language processing to interpret structure). An approach based on spatial molecular geometry in Cartesian space aligns with the well-established practice of using this type of molecular representation in computational chemistry with popular force field and DFT methods. We further differentiate the utility of coordinate-based representations in our work due to the observation that the common Coulomb matrix representation does not distinguish the chirality of molecules. In addition, the simplicity of the encoding allows for easy analysis of molecular structure without having to result in more complex graph-based representations and corresponding neural networks.^26,27,56^

### Data Analysis

**Figure 4:**
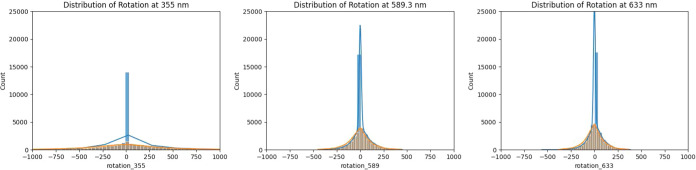
Distribution of calculated optical rotations for all molecules from classes {0} and {1} at the 3 wavelengths. The blue line represents the fit of the distribution for class {0}, and the orange line represents the fit of the distribution for class {1} after filtering outliers *z* > 3.

Because this work required the calculation of the specific rotations for over a hundred thousand organic molecules using DFT, the availability of these data allowed for some minimal statistical analysis of the distributions. Using the class {0} and class {1} molecules with optical rotations calculated at 355, 589.3, and 633 nm, distributions of the specific rotations can be analyzed and compared. Figure 4 shows histograms for the distributions of the class {0} and {1} data, and Table 5 shows a summary of the descriptive parameters for the normal distributions of the calculated values.

**Table 5:** Summary of Normal Distribution Parameters for the Three Wavelengths for Molecular Classes {0} and {1}

molecular class	wavelength (nm)	mean	Std. Dev.	skewness	kurtosis
{0}	355	30.7	987.6	1.476	196.92
	589	7.8	94.6	0.136	4.22
	633	6.7	79.5	0.133	4.13
{1}	355	–17.4	806.0	0.041	18.37
	589	–6.3	129.6	–0.069	1.08
	633	–5.3	108.9	–0.068	1.00

As expected, there are significantly more molecules with zero chiral centers that are predicted to have optical rotations closer to 0 at each of the wavelengths. The majority of the optical rotations for molecules with one chiral center has nonzero values, whereas the majority of optical rotation degrees for molecules with zero chiral centers is near 0. The histograms demonstrate that molecules with one chiral center have a much broader distribution across a range of optical rotations. It should be noted that the DFT calculation of optical rotation is performed at a fixed geometry in the gas phase, and effects from different conformational configurations, vibrations, and internal rotations are not considered here. Each molecular representation from the QM9 data set is treated as a unique molecule.

In all three data sets separated by wavelength, there is less than one percent of outliers whose absolute degree of rotation is greater than 1000 degrees. The absolute value of a single outlier can be as high as 100,000 degrees, which usually corresponds to the calculation of the optical rotation at a wavelength that is very close in energy to an excitation as predicted by the DFT model.

Calculation of the normal distribution parameters for the molecular classes {0} and {1} shows that the optical rotations are centered near 0 and that the standard deviations increase at shorter wavelengths. This is likely due to the larger variation in the optical rotation values at higher frequencies for many of the organic molecules that have excitations at shorter wavelengths. The kurtosis for the distribution of optical rotations for the molecules in class {0} is higher than the one for the distribution of optical rotations for molecules in class {1} which is consistent with the observation that molecules with zero chiral centers are more likely to be centered around a zero optical rotation. Finally, there is no correlation between the (R)/(S) of a molecule and the sign of the optical rotation. Using scipy’s Pearson correlation coefficient and p-value for testing noncorrelation, as expected, there is only a −0.0003 correlation between (R)/(S) configuration and optical rotation sign with a p-value of 0.96.

### Machine Learning

The OHECC representations of the molecules, along with their labels, as provided by RDKit and Gaussian, were used to train supervised learning models using random forest, XGBoost, and artificial neural network algorithms. In the tables showing the confusion matrices, downsampling may be applied to count for class imbalance, resulting in a different number of records from the original data set as presented in Table 1. Downsampling was chosen to address the class imbalance problem since it avoids the difficulty of having to repeatedly select or generate molecules from the minority class and avoids overfitting of the models from repeated selections in the data set.^57^

First, we examine the existence of zero or one chiral centers and the existence of zero or nonzero chiral centers using binary classification. In order to analyze the results of the machine learning models, confusion matrices for each of the classification models along with the labeled experiments are presented in Tables 6 and 7. In each case, the first row represents the true values, and the left column represents the predicted values. The diagonals represent the number of correct classifications, while the off-diagonals represent false positives or false negatives. All three algorithms are relatively successful at being able to distinguish a molecule from having no chiral centers with others that have one chiral center or even more than one chiral centers.

**Table 6:** Confusion Matrices for Three Machine Learning Models for Binary Classification of 0 vs 1 Chiral Centers^a^

random forest			XGBoost			neural network		
	{0}	{1}		{0}	{1}		{0}	{1}
{0}	3781	727	{0}	3967	539	{0}	3820	722
{1}	481	4011	{1}	406	4088	{1}	636	3822

a Row labels represent predicted classifications, and column labels represent true classifications.

**Table 7:** Confusion Matrices for Three Machine Learning Models for Binary Classification of 0 vs 1, 2, or More Chiral Centers^a^

random forest			XGBoost			neural network		
	{0}	{0}^C^		{0}	{0}^C^		{0}	{0}^C^
{0}	6424	750	{0}	6747	487	{0}	6593	621
{0}^C^	648	6578	{0}^C^	522	6644	{0}^C^	759	6427

a Row labels represent predicted classifications, and column labels represent true classifications. {0}^C^ represents the complement of the set containing molecules with 0 chiral centers, i.e., all molecules containing one or more chiral centers in the filtered QM9 data set.

Machine learning has also been used to classify the CIP labels (R,S) for a molecule with one chiral center. Additionally, machine learning has been used to predict the calculated sign of the optical rotation at 589.3 nm for molecules with one chiral center and then again for all molecules with any number of chiral centers. Confusion matrices for these studies with the respective machine learning models are provided in Tables 8, 9, and 10.

**Table 8:** Confusion Matrices for Three Machine Learning Models for Classification of (R) vs (S)^a^

random forest			XGBoost			neural network		
	(R)	(S)		(R)	(S)		(R)	(S)
(R)	1619	624	(R)	1960	599	(R)	1580	681
(S)	578	1774	(S)	543	1763	(S)	604	1730

a Row labels represent predicted classifications, and column labels represent true classifications

**Table 9:** Confusion Matrices for Three Machine Learning Models for Classification of – vs + for Molecules with One Chiral Center^a^

random forest			XGBoost			neural network		
	–	+		–	+		–	+
–	1808	599	–	1760	647	–	1582	772
+	708	1470	+	656	1522	+	835	1396

a Row labels represent predicted classifications, and column labels represent true classifications.

**Table 10:** Confusion Matrices for Three Machine Learning Models for Classification of – vs + for All Molecules^a^

random forest			XGBoost			neural network		
	–	+		–	+		–	+
–	8751	3331	–	8640	3360	–	7558	4381
+	3444	8268	+	3514	8280	+	4439	7416

a Row labels represent predicted classifications, and column labels represent true classifications.

A summary of the performance of each of the binary classifications is presented in Table 11. The results in the table demonstrate that machine learning can be successful for classifying molecules based on chiral structure and properties. The accuracy, precision, and recall observed are successful for what is expected typically from machine learning algorithms with relatively complex data. It appears that XGBoost provides the highest *F*_1_ score for three out of the five tasks and the best accuracies for four out of five tasks. XGBoost has been shown to be a reliable machine learning algorithm for tabular data when the data set size is relatively limited.^32^ The simple neural networks did not perform as well relative to the other methods likely because there is not enough data for the method to work as well as it could.^58^

**Table 11:** Comparison of Machine Learning Algorithms for Binary Classifications of Chiral Properties

chiral property	molecular classes	method	accuracy	precision	recall	*F*_1_
chiral existence	{0, 1, ···, 8}	RF	0.903	0.896	0.908	0.902
		XGBoost	0.930	0.933	0.928	0.930
		ANN	0.904	0.914	0.897	0.905
0 vs 1 chiral center	{0, 1}	RF	0.866	0.839	0.887	0.862
		XGBoost	0.895	0.880	0.907	0.894
		ANN	0.849	0.841	0.857	0.849
R vs S	{1}	RF	0.738	0.722	0.737	0.729
		XGBoost	0.752	0.738	0.757	0.748
		ANN	0.720	0.699	0.723	0.711
+ vs –	{1}	RF	0.715	0.751	0.719	0.735
		XGBoost	0.716	0.731	0.729	0.730
		ANN	0.650	0.672	0.655	0.663
+ vs – (All)	{0, 1, ···, 8}	RF	0.715	0.724	0.718	0.721
		XGBoost	0.711	0.720	0.711	0.715
		ANN	0.629	0.633	0.630	0.632

It is observed that predictions of chiral center existence are more accurate compared to predictions of the (R) or (S) configuration or the sign of the optical rotation. This may suggest that the OHECC representation may be able to help the machine learning models identify molecular symmetry relatively easily. It is interesting to note that the accuracy of the prediction of the sign of the optical rotation is not far from the accuracy to predict properties based on pure configuration of the molecule (e.g., chiral center existence and CIP label). This observation suggests that machine learning can determine the sign of rotation based on the structural configuration of the molecule. The model inputs include the position and type of atoms, and it appears that this information is sufficient to train a model that has a better probability of predicting the sign of rotation than random guesses. This implies that the sign of optical rotation is strongly influenced by the structural arrangement of the atoms or the machine learning models are able to elucidate some quantum chemical behavior that allows for a prediction that is not random or unreliable.

For predicting the sign of the optical rotation at 589.3 nm, it is important to note that the labeled data are calculated using DFT. It is known that there may be inaccuracies in the calculation of optical rotation and that these machine learning models are limited in modeling the DFT calculated optical rotation values rather than any values that are measured experimentally. Using a large basis set, Stephens et al. reported the same sign for 27 out of 28 molecules where experimental data was available for comparison.^11^ In general, the accuracies are better for rigid molecules.

In order to determine the capability of these machine learning models to predict the number of chiral centers based on the OHECC representation, a multilevel classification can be used. Table 12 demonstrates that machine learning labeling of the molecules based on the number of chiral centers also works well. While there are a few misclassifications, the models are able to determine the number of chiral centers in a molecule for most of the data set, as evidenced by the larger numbers in the diagonals of the confusion matrices for each of the machine learning models. Note that the number of predicted chiral centers is limited up to 4 in this case due to the relatively small number of molecules in the data set that have 5 or more chiral centers. Of course, tools like RDKit can provide this labeling with better accuracy, but the goal of this classification is to show the possibilities that machine learning may provide for classifying molecules based on the molecule’s atomic positions.

**Table 12:** Confusion Matrices for Three Machine Learning Models for Multiclassification of 0, 1, 2, etc. Chiral Centers^a^

random forest						XGBoost						neural network					
	{0}	{1}	{2}	{3}	{4}		{0}	{1}	{2}	{3}	{4}		{0}	{1}	{2}	{3}	{4}
{0}	1588	232	98	29	13	{0}	1731	783	70	36	6	{0}	1435	303	143	44	38
{1}	268	1420	223	105	36	{1}	249	1397	220	73	15	{1}	252	1176	353	149	44
{2}	88	348	1175	212	154	{2}	64	293	1293	236	82	{2}	129	359	1042	326	154
{3}	45	103	270	1358	253	{3}	15	77	225	1503	220	{3}	43	155	371	1166	332
{4}	25	37	96	290	1534	{4}	8	20	97	202	1703	{4}	20	47	133	384	1402

a Row labels represent predicted classifications, and column labels represent true classifications.

For molecules whose labels are incorrectly predicted, it is observed that the trained model predicts the labels adjacent to the true labels. For example, if a molecule has two chiral centers but the prediction is wrong by the model, then it is likely that the model will give a prediction of 1 or 3 chiral centers rather than much fewer or much greater possible numbers of chiral centers.

For each of the machine learning models, the necessary class of molecules needed for training was sampled at random. One variable in this procedure that leads to additional investigation is the relative size (or number of atoms) in a molecule that is used for training compared to the number of atoms in a molecule that was used for inference. It is assumed that molecules of similar size should be used for training a model and that models trained with similar-sized molecules should be used for classifying unknown molecules.

To maintain a decent proportion between large and small molecules, we set a boundary of 17 atoms in separating small and large molecules. If a molecule from the QM9 data set has 17 or fewer atoms, then we regard this molecule as a small molecule during the machine learning process. In summary, we have 33,498 small molecules with 23,930 from class {0} and 25,650 large molecules with 12,245 from class {0}. Because of the relatively fast performance of the random forest algorithm, this method was used to test the size dependence of the molecules on classification.

We noticed that if we use a training set consisting of small molecules to fit a testing set of similar-sized small molecules, we obtain a higher accuracy at around 90% compared to using a set of small molecules for training to fit a testing set containing large molecules. This result may suggest that the properties found in small molecules by our model may not be representative enough to make chirality predictions for larger molecules. The better results obtained with smaller molecules may be the result of a larger portion of the OHECC molecular representation being used to describe the area of the molecule that represents the chiral center and the attached substituents, whose positions are modified depending on the chirality. In this case, the part of the representation that influences the chirality of the molecule may provide a larger “signal-to-noise” ratio compared with larger molecules, where the functional part is a small percentage of the overall volume of space the molecule occupies. Finally, training a model with small molecules and validating with big molecules provides an accuracy of around 72% for the validation. This suggests that using larger molecules to train a model for use in inferencing with larger molecules will result in a reduction of accuracy.

The analysis presented here suggests that the size of molecules is an important consideration when building machine learning models based on inputs from Cartesian coordinates for the properties considered in this work. Of course, there are a limited number of smaller molecules, and this can affect the quality of certain machine learning methods that may require larger data sets for better fits. In this work, we have explored a subset of the QM9 data set with about 121,000 molecules. The data set examined in this work contains only C, H, O, N, and F atoms. Additional data sets with a greater number of molecules are also available. The QM9 data set is a small subset from GDB-17 database, which is a collection of all possible molecules made up of under 17 atoms.^59^ Future work may involve examining molecules sampled from the GDB-17 chemical data set, which contains over 166 billion molecules. The larger data set will also allow further exploration of the effect of molecular size on machine learning accuracy. In addition to working with larger data sets, regression models may also be used for predicting specific rotations.

## Conclusions

Machine learning algorithms can be used to predict chiral properties and signs of optical rotation, as determined by quantum chemistry software based on density functional theory. While there is room for improvement in the accuracy, the fact that these algorithms can perform better than pure statistical odds is a sign that they are extracting features from the absolute spatial information on the atoms and their identity to learn an inherent chiral property of a molecule. Of course, DFT approximations and errors are real, and only the most accurate machine learning models can be constructed from experimental data. Although the more traditional random forest algorithm performed slightly better compared to the XGBoost and neural network models, the accuracy of the models can be viewed as being relatively similar. As a result, data representation for the molecules is likely more important than the machine learning algorithm chosen.

This work has demonstrated that molecular representations based on Cartesian coordinates are valid representations for machine learning models that are highly dependent on the structural configuration of molecules. Our choice of representation is based on the principle that simple models and simple features should be used (ideally) for applications of machine learning. The goal of the present work is to show that machine learning is a viable tool and a worthwhile pursuit for making predictions of chiral properties based on the results from a set of molecules from the QM9 data set. The results presented here demonstrate that machine learning can be used to classify molecules based on the atom type and position alone, whether that is a relatively simple task such as the number of chiral centers or a more challenging one, such as the sign of optical rotation. While the results obtained are reasonable for initial explorations of the application of machine learning to molecular properties, additional work will be required to obtain more accurate results and to explore additional possibilities and applications to chemistry.

## Data Availability

InChI and OHECC representations and stereochemical information for the molecules used in this study are available at https://zenodo.org/doi/10.5281/zenodo.13380412. A Jupyter notebook demonstrating how to extract molecules from the NumPy file containing the data is provided at https://github.com/bcmort/OHECC.

## References

[ref1] Frisch M. J.; Trucks G. W.; Schlegel H. B.; Scuseria G. E.; Robb M. A.; Cheeseman J. R.; Scalmani G.; Barone V.; Petersson G. A.; Nakatsuji H. Gaussian 16 Revision C.01; Gaussian Inc.: Wallingford CT, 2016.

[ref2] Aprá E.; Bylaska E. J.; de Jong W. A.; Govind N.; Kowalski K.; Straatsma T. P.; Valiev M.; van Dam H. J. J.; Alexeev Y.; Anchell J.; et al. NWChem: Past, and future. J. Chem. Phys. 2020, 152, 184102 10.1063/5.0004997. 32414274

[ref3] Aidas K.; Angeli C.; Bak K. L.; Bakken V.; Bast R.; Boman L.; Christiansen O.; Cimiraglia R.; Coriani S.; Dahle P.; et al. The Dalton quantum chemistry program system. WIREs Comput. Mol. Sci. 2014, 4, 269–284. 10.1002/wcms.1172. PMC4171759 25309629

[ref4] Epifanovsky E.; Gilbert A. T. B.; Feng X.; Lee J.; Mao Y.; Mardirossian N.; Pokhilko P.; White A. F.; Coons M. P.; Dempwolff A. L.; et al. Software for the frontiers of quantum chemistry: An overview of developments in the Q-Chem 5 package. J. Chem. Phys. 2021, 155, 084801 10.1063/5.0055522. 34470363 PMC9984241

[ref5] Biot J.-B. Phénomènes de polarisation successive, observés dans des fluides homogènes. Bull. Soc. Philomath 1815, 190, 1815.

[ref6] Pasteur L. Memoir on the relationship that can exist between crystalline form and chemical composition, and on the cause of rotary polarization. CR Acad. Sci. Paris 1848, 26, 535–538.

[ref7] Cotton A. Absorption inégale des rayons circulaires droit et gauche dans certains corps actifs. Compt. Rend. 1895, 120, 989–991.

[ref8] Mason S. F. Optical Activity and Chiral Discrimination; Springer Science & Business Media, 2013; Vol. 48.

[ref9] Liu Y.; Wu Z.; Armstrong D. W.; Wolosker H.; Zheng Y. Detection and analysis of chiral molecules as disease biomarkers. Nat. Rev. Chem. 2023, 7, 355–373. 10.1038/s41570-023-00476-z. 37117811 PMC10175202

[ref10] Polavarapu P. L. Ab initio molecular optical rotations and absolute configurations. Mol. Phys. 1997, 91, 551–554. 10.1080/00268979709482744.

[ref11] Stephens P. J.; Devlin F. J.; Cheeseman J. R.; Frisch M. J. Calculation of optical rotation using density functional theory. J. Phys. Chem. A 2001, 105, 5356–5371. 10.1021/jp0105138.

[ref12] Stephens P. J.; Devlin F. J.; Cheeseman J. R.; Frisch M. J.; Rosini C. Determination of absolute configuration using optical rotation calculated using density functional theory. Org. Lett. 2002, 4, 4595–4598. 10.1021/ol0201714. 12489938

[ref13] Stephens P. J.; Pan J. J.; Devlin F. J.; Cheeseman J. R. Determination of the absolute configurations of natural products using TDDFT optical rotation calculations: The iridoid oruwacin. J. Nat. Prod. 2008, 71, 285–288. 10.1021/np070502r. 18211006

[ref14] Stephens P.; McCann D.; Cheeseman J.; Frisch M. Determination of absolute configurations of chiral molecules using ab initio time-dependent density functional theory calculations of optical rotation: How reliable are absolute configurations obtained for molecules with small rotations?. Chirality 2005, 17, S52–S64. 10.1002/chir.20109. 15747317

[ref15] Crawford T. D.; Owens L. S.; Tam M. C.; Schreiner P. R.; Koch H. Ab initio calculation of optical rotation in (P)-(+)-[4]triangulane. J. Am. Chem. Soc. 2005, 127, 1368–1369. 10.1021/ja042787p. 15686357

[ref16] Crawford T. D.; Tam M. C.; Abrams M. L. The current state of ab initio calculations of optical rotation and electronic circular dichroism spectra. J. Phys. Chem. A 2007, 111, 12057–12068. 10.1021/jp075046u. 17985851

[ref17] Crawford T. D.; Stephens P. J. Comparison of time-dependent density-functional theory and coupled cluster theory for the calculation of the optical rotations of chiral molecules. J. Phys. Chem. A 2008, 112, 1339–1345. 10.1021/jp0774488. 18198852

[ref18] Srebro-Hooper M.; Autschbach J. Calculating natural optical activity of molecules from first principles. Annu. Rev. Phys. Chem. 2017, 68, 399–420. 10.1146/annurev-physchem-052516-044827. 28463650

[ref19] Carleo G.; Cirac I.; Cranmer K.; Daudet L.; Schuld M.; Tishby N.; Vogt-Maranto L.; Zdeborová L. Machine learning and the physical sciences. Rev. Mod. Phys. 2019, 91, 045002 10.1103/RevModPhys.91.045002.

[ref20] Greener J. G.; Kandathil S. M.; Moffat L.; Jones D. T. A guide to machine learning for biologists. Nat. Rev. Mol. Cell Biol. 2022, 23, 40–55. 10.1038/s41580-021-00407-0. 34518686

[ref21] Rupp M.; Tkatchenko A.; Müller K.-R.; von Lilienfeld O. A. Fast and accurate modeling of molecular atomization energies with machine learning. Phys. Rev. Lett. 2012, 108, 058301 10.1103/PhysRevLett.108.058301. 22400967

[ref22] Faber F. A.; Hutchison L.; Huang B.; Gilmer J.; Schoenholz S. S.; Dahl G. E.; Vinyals O.; Kearnes S.; Riley P. F.; von Lilienfeld O. A. Prediction errors of molecular machine learning models lower than hybrid DFT error. J. Chem. Theory Comput. 2017, 13, 5255–5264. 10.1021/acs.jctc.7b00577. 28926232

[ref23] Schütt K. T.; Gastegger M.; Tkatchenko A.; Müller K.-R.; Maurer R. J. Unifying machine learning and quantum chemistry with a deep neural network for molecular wavefunctions. Nat. Commun. 2019, 10, 5024 10.1038/s41467-019-12875-2. 31729373 PMC6858523

[ref24] Mamede R.; de Almeida B. S.; Chen M.; Zhang Q.; de Sousa J. A. Machine learning classification of one-chiral-center organic molecules according to optical rotation. J. Chem. Inf. Model. 2021, 61, 67–75. 10.1021/acs.jcim.0c00876. 33350814

[ref25] Vermeyen T.; Cunha A.; Bultinck P.; Herrebout W. Impact of conformation and intramolecular interactions on vibrational circular dichroism spectra identified with machine learning. Commun. Chem. 2023, 6, 148 10.1038/s42004-023-00944-z. 37438485 PMC10338531

[ref26] Schütt K. T.; Kindermans P.-J.; Sauceda H. E.; Chmiela S.; Tkatchenko A.; Müller K.-R. SchNet: A Continuous-Filter Convolutional Neural Network for Modeling Quantum Interactions. 2017, arXiv:1706.08566. arXiv.org e-Printarchive. https://arxiv.org/abs/1706.08566.

[ref27] Liu Y. L.; Wang Y.; Vu O.; Moretti R.; Bodenheimer B.; Meiler J.; Derr T. In Interpretable Chirality-Aware Graph Neural Network for Quantitative Structure Activity Relationship Modeling in Drug Discovery, Proceedings of the AAAI Conference on Artificial Intelligence; AAAI, 2023; pp 14356–14364.

[ref28] Goodfellow I.; Bengio Y.; Courville A. Deep Learning; MIT Press, 2016 http://www.deeplearningbook.org.

[ref29] DeGroot M. H.; Schervish M. J. Probability and Statistics; Addison-Wesley, 2012.

[ref30] Ho T. K. In Random Decision Forests, Proceedings of 3rd International Conference on Document Analysis and Recognition; IEEE, 1995; pp 278–282.

[ref31] Breiman L. Random forests. Mach. Learn. 2001, 45, 5–32. 10.1023/A:1010933404324.

[ref32] Chen T.; Guestrin C. In XGBoost, Proceedings of the 22nd ACM SIGKDD International Conference on Knowledge Discovery and Data Mining; ACM Digital Library, 2016.

[ref33] https://github.com/dmlc/xgboost.

[ref34] McCulloch W. S.; Pitts W. A logical calculus of the ideas immanent in nervous activity. Bull. Math. Biol. 1943, 5, 115–133. 10.1007/BF02478259. 2185863

[ref35] Polavarapu P. L.; Zhao C. Ab initio predictions of anomalous optical rotatory dispersion. J. Am. Chem. Soc. 1999, 121, 246–247. 10.1021/ja9828556.

[ref36] Ruud K.; Helgaker T. Optical rotation studied by density-functional and coupled-cluster methods. Chem. Phys. Lett. 2002, 352, 533–539. 10.1016/S0009-2614(01)01492-0.

[ref37] Becke A. D. Density-functional thermochemistry. III. The role of exact exchange. J. Chem. Phys. 1993, 98, 5648–5652. 10.1063/1.464913.

[ref38] Lee C.; Yang W.; Parr R. G. Development of the Colle-Salvetti correlation-energy formula into a functional of the electron density. Phys. Rev. B 1988, 37, 785–789. 10.1103/PhysRevB.37.785. 9944570

[ref39] Vosko S. H.; Wilk L.; Nusair M. Accurate spin-dependent electron liquid correlation energies for local spin density calculations: A critical analysis. Can. J. Phys. 1980, 58, 1200–1211. 10.1139/p80-159.

[ref40] Stephens P. J.; Devlin F. J.; Chabalowski C. F.; Frisch M. J. Ab initio calculation of vibrational absorption and circular dichroism spectra using density functional force fields. J. Phys. Chem. A 1994, 98, 11623–11627. 10.1021/j100096a001.

[ref41] Ditchfield R.; Hehre W. J.; Pople J. A. Self-consistent molecular-orbital methods. IX. An extended gaussian-type basis for molecular-orbital studies of organic molecules. J. Chem. Phys. 1971, 54, 724–728. 10.1063/1.1674902.

[ref42] Hariharan P. C.; Pople J. A. The influence of polarization functions on molecular orbital hydrogenation energies. Theor. Chim. Acta 1973, 28, 213–222. 10.1007/BF00533485.

[ref43] Hehre W. J.; Ditchfield R.; Pople J. A. Self-consistent molecular orbital methods. XII. Further extensions of gaussian-type basis sets for use in molecular orbital studies of organic molecules. J. Chem. Phys. 1972, 56, 2257–2261. 10.1063/1.1677527.

[ref44] Yanai T.; Tew D. P.; Handy N. C. A new hybrid exchange–correlation functional using the Coulomb-attenuating method (CAM-B3LYP). Chem. Phys. Lett. 2004, 393, 51–57. 10.1016/j.cplett.2004.06.011.

[ref45] Zhou Y.; Mort B. QM9-OR: DFT optimized geometries and optical rotations for selected QM9 molecules. 2024 10.5281/zenodo.13380412.

[ref46] Cahn R. S.; Ingold C. K.; Prelog V. The specification of asymmetric configuration in organic chemistry. Experientia 1956, 12, 81–94. 10.1007/BF02157171.

[ref47] Pedregosa F.; Varoquaux G.; Gramfort A.; Michel V.; Thirion B.; Grisel O.; Blondel M.; Prettenhofer P.; Weiss R.; Dubourg V.; et al. Scikit-learn: Machine learning in Python. J. Mach. Learn. Res. 2011, 12, 2825–2830.

[ref48] Paszke A.; Gross S.; Massa F.; Lerer A.; Bradbury J.; Chanan G.; Killeen T.; Lin Z.; Gimelshein N.; Antiga L. Advances in Neural Information Processing Systems 32; Curran Associates, Inc., 2019; pp 8024–8035.

[ref49] https://github.com/szagoruyko/pytorchviz.

[ref50] Ghosh K.; Stuke A.; Todorović M.; Jørgensen P. B.; Schmidt M. N.; Vehtari A.; Rinke P. Deep learning spectroscopy: Neural networks for molecular excitation spectra. Adv. Sci. 2019, 6, 1801367 10.1002/advs.201801367. PMC6498126 31065514

[ref51] Weininger D. SMILES, a chemical language and information system. 1. Introduction to methodology and encoding rules. J. Chem. Inf. Comput. Sci. 1988, 28, 31–36. 10.1021/ci00057a005.

[ref52] Pinheiro G. A.; Mucelini J.; Soares M. D.; Prati R. C.; Da Silva J. L. F.; Quiles M. G. Machine learning prediction of nine molecular properties based on the SMILES representation of the QM9 quantum-chemistry dataset. J. Phys. Chem. A 2020, 124, 9854–9866. 10.1021/acs.jpca.0c05969. 33174750

[ref53] Wang S.; Guo Y.; Wang Y.; Sun H.; Huang J. In Smiles-Best, Proceedings of the 10th ACM International Conference on Bioinformatics, Computational Biology and Health Informatics. New York, NY, USA; ACM, 2019.

[ref54] Heller S.; McNaught A.; Stein S.; Tchekhovskoi D.; Pletnev I. InChI - The worldwide chemical structure identifier standard. J. Cheminform. 2013, 5, 7 10.1186/1758-2946-5-7. 23343401 PMC3599061

[ref55] RDKit: Open-Source Cheminformatics Software. https://www.rdkit.org.

[ref56] Pattanaik L.; Ganea O.-E.; Coley I.; Jensen K. F.; Green W. H.; Coley C. W. Message Passing Networks for Molecules with Tetrahedral Chirality. 2020, arXiv:2012.00094. arXiv.org e-Printarchive. https://arxiv.org/abs/2012.00094.

[ref57] He H.; Garcia E. A. Learning from Imbalanced Data. IEEE Trans. Knowl. Data Eng. 2009, 21, 1263–1284. 10.1109/TKDE.2008.239.

[ref58] Alwosheel A.; van Cranenburgh S.; Chorus C. G. Is your dataset big enough? Sample size requirements when using artificial neural networks for discrete choice analysis. J. Choice Model. 2018, 28, 167–182. 10.1016/j.jocm.2018.07.002.

[ref59] Ramakrishnan R.; Dral P. O.; Rupp M.; von Lilienfeld O. A. Quantum chemistry structures and properties of 134 kilo molecules. Sci. Data 2014, 1, 140022 10.1038/sdata.2014.22. 25977779 PMC4322582

